# Genetic mapping and identification of QTL for earliness in the globe artichoke/cultivated cardoon complex

**DOI:** 10.1186/1756-0500-5-252

**Published:** 2012-05-23

**Authors:** Ezio Portis, Davide Scaglione, Alberto Acquadro, Giovanni Mauromicale, Rosario Mauro, Steven J Knapp, Sergio Lanteri

**Affiliations:** 1 Di.Va.P.R.A. Plant Genetics and Breeding, University of Torino, via L. da Vinci 44, I-10095, Grugliasco, Torino, Italy; 2 Dipartimento di Scienze delle Produzioni Agrarie e Alimentari (DISPA) – sez. Scienze Agronomiche, University of Catania, via Valdisavoia 5, I-95123, Catania, Italy; 3 Department of Crop and Soil Sciences and Center for Applied Genetic Technologies, University of Georgia, 111 Riverbend Rd, 30602, Athens, GA, USA; 4 Monsanto Company, Woodland, CA, USA

**Keywords:** Cynara cardunculus, Linkage map, Microsatellite, QTL, Earliness

## Abstract

**Background:**

The Asteraceae species *Cynara cardunculus* (*2n* = *2x* = 34) includes the two fully cross-compatible domesticated *taxa* globe artichoke (var. *scolymus* L.) and cultivated cardoon (var. *altilis* DC). As both are out-pollinators and suffer from marked inbreeding depression, linkage analysis has focussed on the use of a two way pseudo-test cross approach.

**Results:**

A set of 172 microsatellite (SSR) loci derived from expressed sequence tag DNA sequence were integrated into the reference *C. cardunculus* genetic maps*,* based on segregation among the F_1_ progeny of a cross between a globe artichoke and a cultivated cardoon. The resulting maps each detected 17 major linkage groups, corresponding to the species’ haploid chromosome number. A consensus map based on 66 co-dominant shared loci (64 SSRs and two SNPs) assembled 694 loci, with a mean inter-marker spacing of 2.5 cM. When the maps were used to elucidate the pattern of inheritance of head production earliness, a key commercial trait, seven regions were shown to harbour relevant quantitative trait loci (QTL). Together, these QTL accounted for up to 74% of the overall phenotypic variance.

**Conclusion:**

The newly developed consensus as well as the parental genetic maps can accelerate the process of tagging and eventually isolating the genes underlying earliness in both the domesticated *C. cardunculus* forms. The largest single effect mapped to the same linkage group in each parental maps, and explained about one half of the phenotypic variance, thus representing a good candidate for marker assisted selection.

## Background

The Asteraceae (ex Compositae) species *Cynara cardunculus* L. comprises three *taxa*, namely the two domesticated form globe artichoke (var. *scolymus*) and cultivated cardoon (var. *altilis*), along with their common ancestor the wild cardoon (var. *sylvestris*). While the globe artichoke was selected for its large immature inflorescences, the cardoon was selected for its fleshy leaves and stalks. The three *taxa* remain fully cross-compatible with one another, and their F_1_ hybrids are fertile. The species complex has a highly heterozygous diploid genome (2*n* = 2*x* = 34), maintained by its cross-pollinating habit [1]. The domesticated forms produce a variety of nutraceuticals and pharmaceutically active compounds like inulin, mono- and di-caffeoylquinic acids [2-6] and sesquiterpene lactones, which are responsible for its characteristic bitterness [7-9]. Globe artichoke contributes significantly to the Mediterranean agricultural economy in the form of an annual production of ~750Mt worth over US$500 M annually. It is also cultivated in the Americas, North Africa and China (http://faostat.fao.org).

Most of the Mediterranean globe artichoke germplasm is vegetatively propagated, and a number of varietal groups have been defined on the basis of the appearance of the inflorescence and harvesting time of the head (*capitula*) Flowering can be induced between autumn and spring in early flowering types by watering dormant underground shoots, whereas late flowering types flower only during spring and early summer. A common breeding target for both vegetatively and seed-propagated varieties is the promotion of earliness since inflorescences produced in the early part of the year command a higher price than those produced in the summer. Unlike globe artichoke, the cultivated cardoon is exclusively seed-propagated, and is generally handled as an annual crop. Of late it has been promoted as a source of lignocellulosic biomass [10-12] and the evidence suggests that it should be possible to derive types able to flower early, to produce stems with a high lignin content and to generate biomass with a good level of energy efficiency [13,14]. Earliness is therefore an important trait in both domesticated forms.

The first generation of *C. cardunculus* marker-based genetic maps [15-17] have resulted in a cultivated cardoon map composed of nearly 200 loci (17 major linkage groups, LGs) spanning just over 10 M, and a globe artichoke one featuring 326 loci (20 major LGs) spanning about 15 M. The two maps have since been integrated on the basis of common loci with the inclusion of a number of genes involved in the synthesis of caffeoylquinic acids [18,19]. More recently crosses between globe artichoke and its ancestor wild cardoon have generated highly segregating F_1_ populations exploitable as ornamentals [20] as well as for mapping studies [21].

The multi-allelism of many microsatellite (SSR) loci makes them particularly well suited as bridging markers to link independent maps. The design of SSR assays requires DNA sequence, which in globe artichoke exists at present largely in the form of expressed sequence tag (EST) sequence (http://compgenomics.ucdavis.edu/). Over 4,000 potential EST-SSR loci have been identified from this sequence resource, and the experimental testing of a sample of 300 loci showed that more than one half were informative between the parents of our two mapping populations [22]. In the present report, we describe the integration into the globe artichoke and cultivated cardoon maps of a large number of these EST-SSR loci, and show that they can be used as bridging markers to merge the two maps. The resulting dense maps was then used to identify a number of quantitative trait loci (QTL) underlying early head production in *C. cardunculus.*

## Results and discussion

### Genotyping

Six of the 178 informative *Cynara Expressed Microsatellite* (CyEM) markers, identified by Scaglione et al. [22], were excluded from the analysis on the basis of excessive missing values. Of the remaining 172, 54 segregated in both parents (46 as 1:1:1:1, eight as 1:2:1) and 118 in just one of the parents (85 in globe artichoke ‘Romanesco C3’, 33 in cultivated cardoon ‘Altilis 41’). On the whole 228 microsatellite markers were available for map construction (Table 1). Eleven of these loci suffered from mild segregation distortion (χ_α=0.05_^2^ < *χ*^2^ ≤ χ_α=0.01_^2^) but just one (CyEM_58) from severe distortion (*χ*^2^ > χ_α=0.01_^2^). Since CyEM_58 was excluded from the mapping analysis, this left a total of 227 SSR loci. Co-dominant markers appear to be less affected by segregation distortion than dominant ones [23,24], and this certainly was the case for *C. cardunculus*, where ~13% of AFLP and S-SAP loci [17], but only ~5% of SSRs and SNPs are distorted. Segregation distortion has been associated with statistical bias and/or with errors in genotyping, but they can also stem from a number of biological phenomena affecting meiosis, fertilization and embryogenesis [25] as well as the presence of null alleles. Null alleles at SSR loci are not uncommon, as they can arise where either one (or both) of the primers fail to anneal because of sequence mismatch or the deletion of the whole locus, and cause an higher apparent number of homozygotes because they can no longer be distinguished from the heterozygotes [26]. In this situation, the options are either to disregard the affected loci, to score segregation in the same way as for a dominant marker [27], to attempt to redesign the primers [28,29], or to adjust allele frequencies on the basis of a global estimate of the frequency of null alleles. As recently described by Lanteri et al. [20] the null alleles segregating in a Mendelian fashion were identified, thus limiting the segregation distortion in our populations. As noted previously [15,17], although the inclusion of loci distorted at the 1% level and above increases the frequency of type I errors, it does help to maintain marker density throughout the map.

**Table 1:** Polymorphism and segregation patterns for the SSR loci used for map construction

**SR prefix**	**Number of loci**	**Marker type**	**Segregation type < Romanesco C3 x Altilis 41>**				**References**
			**<ab x aa>** **<ab x cc>**	**<aa x ab>** **<aa x bc>**	**<ab x ab>**	**<ab x ac > <ab x cd>**	
CDAT	1	Genic	1	-	-	-	Acquadro et al. [63]
CLIB	3	Inter-genic	2	-	-	1	Acquadro et al. [63]
CMAL	5	Inter-genic	4	1	-	-	Acquadro et al. [64]
CMAFLP	2	Inter-genic	1	-	-	1	Acquadro et al. [65]
CsPal	1	Genic	1	-	-	-	Sonnante et al. [66]
CsLib	1	Inter-genic	1	-	-	-	Sonnante et al. [66]
CELMS	43	Genic and inter-genic^1^	19	4	1	19	Acquadro et al. [16]
**CyEM**	**172**	**Genic**	**85**	**33**	**8**	**46**	**Scaglione et al.**[22]
**Total**	**228**		**114**	**38**	**9**	**67**	

^1^ 27 out of the 61 CELMS microsatellites are reported to be genic SSR as they contain at least one ORF.

The updated ‘Romanesco C3’ map was built from 574 loci (359 AFLPs, 19 S-SAPs, 189 SSRs and seven SNPs), and the ‘Altilis 41’ one from 373 loci (246 AFLPs, 8 S-SAPs, 114 SSRs and five SNPs); of these, 78 (76 SSRs and two SNPs) were in common between the two parental genotypes. The CyEM SSRs were less informative in the cultivated cardoon than in the globe artichoke. Of the 228 assayed SSR loci 189 (83%) segregated in ‘Romanesco C3’, but only 114 (50%) in ‘Altilis 41’ (Table 1). The difference in level of heterozygosity between these parents has been remarked on before [17] and is thought to be a consequence of the sustained vegetative propagation used in globe artichoke, in contrast to the seed propagation applied to the cultivated cardoon, which led to a certain degree of purifying selection aimed at stabilizing production.

### Globe artichoke map

**Figure 1:**
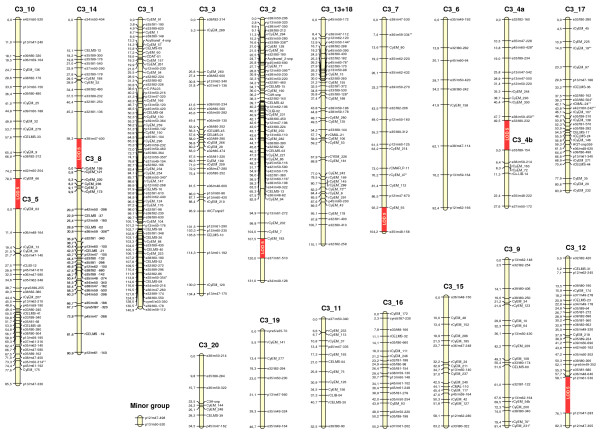
**Genetic map of globe artichoke ‘Romanesco C3’.** Marker names are shown to the right of each LG, with map distances (in cM) to the left. One LG containing <4 loci is labelled as ‘minor group’. Loci showing significant levels of segregation distortion are identified by asterisks (0.1 > *P ≥ 0.05; 0.05 > **P ≥ 0.01). Segments shaded in red indicate where a pair of LGs has merged as a result of reducing the stringency to LOD 5.

The globe artichoke ‘Romanesco C3’ map (LOD 6.0) consisted of 473 loci falling into 20 LGs, each containing at least eight loci (Figure 1). The number of mapped SSRs has now risen from 46 to 185. The largest LG contained 73 loci, and the range in genetic length of the individual LGs was 34.5-140.9 cM. CyEM loci (139 markers) were mapped to all the major LGs, and their inclusion allowed the integration of six AFLP loci which previously had remained unlinked [17]. Two LGs (C3_13 and C3_18) which were previously separated have now been merged, while LG C3_4 has been split into C3_4a and C3_4b as a consequence of more stringent LOD applied (Figure 1). LG C3_17 has increased in genetic length by 36 cM (86%), while that of LG C3_3 and C3_8 has increased by ~30% and ~20%, respectively. The map spanned 1543.8 cM, with a mean inter-marker distance of 3.40 cM, corresponding to a 3.8% increase in length over the earlier map [17], but in a ~28% decrease in the mean inter-marker distance. The proportion of intervals shorter than 7 cM is now 88% (previously 77%), and only six gaps of >15 cM remain. The SSRs appeared to be rather quite uniformly dispersed, although some clustering is present in the distal regions of C3_8, C3_2 and C3_17, and around the putative centromeric region, of C3_3, C3_15 and C3_20. These chromosomal regions are typically enriched for SSRs [30-35]. The relatively low marker saturation present in the distal regions of C3_3 and C3_14 presumably reflects a localized reduced level of polymorphism between the mapping parents.

Some segregation distortion was present at five of the CyEM loci (CyEM_19, _47, _70, _73 and _231; three at α = 0.05 and two at α = 0.01, Figure 1) which affected a cluster of loci on both C3_17 and C3_9. In both cases the distortion was due to an excess of the band detected in the female parent, thus it is likely to have a biological basis, rather than being due to either scoring error or chance [36]. Biological mechanisms causing segregation distortions have been extensively studied in Drosophila [37], and are known to occur in many plant species [38-42]. On the other hand, the other 18 distorted loci were scattered across the genome, a common feature in the genetic maps of both plant and animal species [43].

By lowering the LOD threshold from 6.0 to 5.0, three pairs of LGs were merged: C3_10 with C3_5, C3_14 with C3_8 and C3_4a with C3_4b, resulting in the formation of 17 LGs (corresponding to the haploid chromosome number*,* Figure 1). It also allowed the inclusion of two unlinked pairs of loci (one into C3_2 and the other into C3_12) and the singlet AFLP locus e35/m46-156 (into C3_7). This generated an increase in the genetic length of the map of ~60 cM; one doublet still remains unlinked (Figure 1).

**Figure 2:**
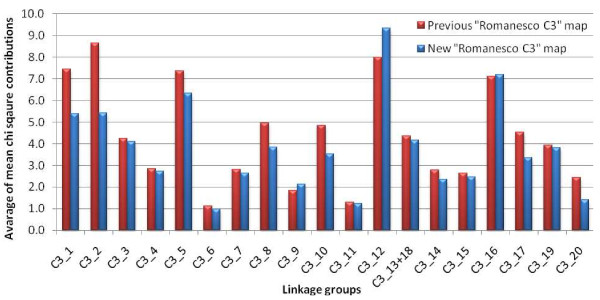
**Variation in the mean goodness-of-fit of markers for each ‘Romanesco C3’ LG**. Variation detected by comparing the current ‘Romanesco C3’ map with that published by Portis et al. [17]. LGs C3_13 and C3_18 have been merged.

Both the goodness-of-fit of marker placement (mean *χ*^2^ contribution) and nearest neighbour fit (cM) were evaluated. Compared to the earlier ‘Romanesco C3’ map [17], the average mean *χ*^2^ contribution of markers across the LGs has been significantly reduced from 5.38 to 4.42 (*t* test at α = 0.005), highlighting the improvement in robustness. The variation in this parameter for each LG is illustrated in Figure 2, which confirms that LGs C3_1, C3_2, C3_5, C3_8, C3_10, C3_17 and C3_20 have all shown an improved goodness-of-fit. C3_12 remained largely composed of AFLP loci (only two CyEM loci were integrated) and thus its robustness was hardly improved. The mean nearest neighbour fit of the CyEM loci (24.3 ± 3.7 cM) was markedly lower (*t* test at α = 0.005) than that of the AFLP loci (51.0 ± 4.6 cM), confirming the desirability of including co-dominant markers to obtain reliable marker placement.

### Cultivated cardoon map

**Figure 3:**
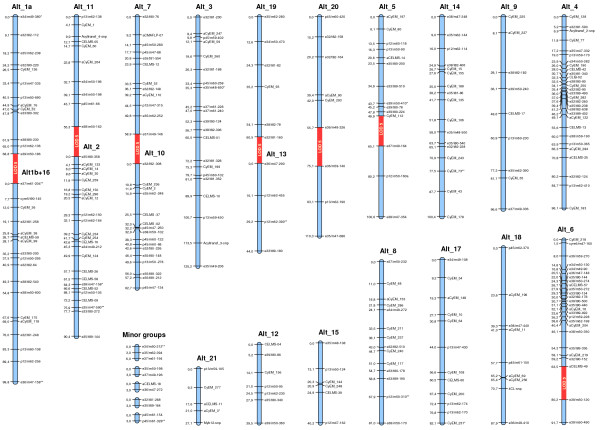
**Genetic map of cultivated cardoon ‘Altilis 41’.** Marker names are shown to the right of each LG, with map distances (in cM) to the left. LGs containing <4 loci are labelled as ‘minor groups’. Loci showing significant levels of segregation distortion are identified by asterisks (0.1 > *P ≥ 0.05; 0.05 > **P ≥ 0.01). Segments shaded in red indicate where a pair of LGs has merged as a result of reducing the stringency to LOD 5.

The genetic map of the cultivated cardoon ‘Altilis 41’ parent (LOD 6.0) was constructed from 373 segregating loci (82 CyEM loci), of which 273 were ordered into 21 major LGs, whose length ranged from 27.1 to 125.2 cM, with the largest LG consisting of 29 loci. The result of integrating the CyEM loci was an increase in the number of major LGs from 17 to 21. This involved the recognition of four new LGs (Alt_18 to _21), the splitting of Alt_1 into two (Alt_1a and Alt_1b) and the merging of Alt_16 and Alt_1b (Figure 3). The updated ‘Altilis 41’ map included 107 SSR loci distributed across all but one (Alt_13) of the major LGs, with a total genetic length of 1485.7 cM and a mean inter-marker distance of 5.44 cM. This represents a marked increase in both length (+42%) and number of loci (+50%), together with a minor decrease in the mean inter-marker distance (−5%). The proportion of intervals smaller than 10 cM (about 80%) was not significantly reduced. Some of the LGs recorded large increases in their genetic length – for example, that of Alt_17 by 64.7 cM, Alt_14 by 58.9 cM and Alt_18 by 57.8 cM. The only LG which recorded a reduction in length was Alt_5. There was some clustering of CyEM loci in the distal region of Alt_2 and around the putative centromeric region of Alt_1b and Alt_15. Three CyEM loci (CyEM_73, _3 and _231; Figure 3) showed a degree of segregation distortion (two at α = 0.05 and one at α = 0.01), but none of these were linked to other distorted loci, similar to the other nine markers showing segregation distortion. The addition of the new SSR markers decreased the mean inter-marker distance on Alt_11 by ~60%, and some gaps in the previous map have been filled; but ten gaps of >15 cM remained, perhaps reflecting regions of genetic fixation which have arisen during cultivated cardoon domestication.

Lowering the LOD threshold to 5.0 led to the merging of four pairs of LG: Alt_1a with Alt_1b, Alt_11 with Alt_2, Alt_7 with Alt_10, and Alt_19 with Alt_13. At this level of stringency the number of LGs corresponded to the haploid chromosome (Figure 3). The lowered stringency also allowed the incorporation of two groups of three linked loci into LGs Alt_20, and Alt_5, and of one doublet into LG Alt_6. As a result, the overall length of the map was increased by 133.5 cM; one triplet and four doublets still remain unlinked (Figure 3).

### The *C. Cardunculus* consensus map

**Figure 4:**
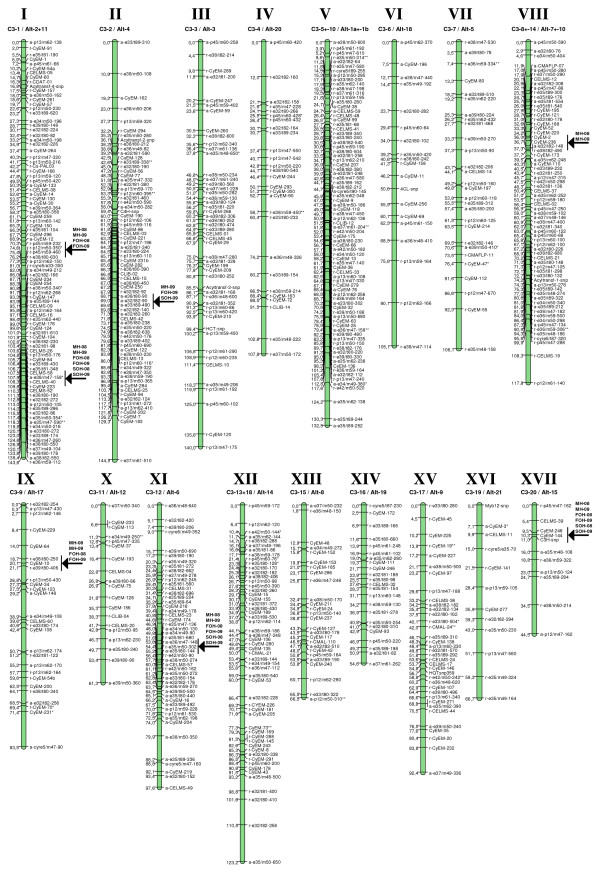
**Consensus genetic map of*C. cardunculus*.** Marker names appear to the right of each LG, with map distances in cM to the left; 'r-' and 'a-' indicate markers segregating only in, respectively, ‘Romanesco C3’ (C3) and ‘Altilis 41’ (Alt41). Arrows indicate the positions of earliness QTL, named as follows: trait abbreviation (MH: main inflorescence; FOH: first order inflorescence; SOH: second order inflorescence) and harvest season (08: “2008”, 09: “2009”).

The number of informative shared co-dominant markers was raised from 19 to 66 (64 SSRs, two SNPs), representing from one to 15 bridging markers per LG. As a result, 19 of the ‘Romanesco C3’ LGs were alignable with 20 of the ‘Altilis 41’ ones (Table 2). There was a one to one correspondence between 18 LG pairs, but C3_1 shared markers with both Alt_11 and Alt_2 (Table 2). C3_4b remained non-aligned, but did harbour a number of SSR loci which were informative for the second step of the analysis; this was not the case for Alt_13 (Table 2). The alignment was followed by the construction of a consensus map based on a LOD threshold of above 5.0 (Figure 4), which succeeded in capturing 694 loci, 227 (217 SSRs, ten SNPs) of which involved co-dominant markers. The map generated 17 LGs with a total genetic length of 1687.6 cM and a mean inter-marker spacing of 2.5 cM; consensus LG numbers (from LG I to LG XVII) have been assigned (Table 2, Figure 4). The length of each individual LG varied from 44.5 to 144.5 cM (mean 99.3 cM), with the largest containing 92 loci. Only three of the CyEM loci (the intercross locus CyEM_134 and the two ‘Altilis 41’ testcross loci CyEM_167 and _79) remained unlinked.

**Table 2:** **Characteristics and alignment of the consensus*C. cardunculus*linkage map**

**Linkage group name**			**Shared markers**	**Size (cM)**	**Total markers**	**Marker density**	**Alignment with globe artichoke x wild cardoon map**^**1**^	
**Consensus LG**	**Romanesco C3**	**Altilis 41**					**Aligned LGs**	**Shared markers**
LG_I	C3_1	Alt_2 + _11	15	143.6	92	1.6	LG_I	21
LG_II	C3_2	Alt_4	10	144.5	72	2.0	LG_II	10
LG_III	C3_3	Alt_3	4	140	50	2.9	LG_III	7
LG_IV	C3_4a + _4b	Alt_20	1	107.8	27	4.1	LG_VI	4
LG_V	C3_5 + _10	Alt_1a + _1b	4	132.5	81	1.7	LG_V and LG_X	14
LG_VI	C3_6	Alt_18	1	105	19	5.8	Mola-18	3
LG_VII	C3_7	Alt_5	2	106.1	28	3.9	LG_VII + LG_XVII	5
LG_VIII	C3_8 + _14	Alt_10 + _7	6	117.8	68	1.8	LG_VIII + LG_XV + tripl.	11
LG_IX	C3_9	Alt_17	7	83.5	27	3.2	LG_IX	5
LG_X	C3_11	Alt_12	2	61.3	19	3.4	LG_XI	6
LG_XI	C3_12	Alt_6	1	97.6	49	2.0	LG_XII	4
LG_XII	C3_(13 + 18)	Alt_14	9	123.2	54	2.3	LG_IV	13
LG_XIII	C3_15	Alt_8	6	66.5	26	2.7	LG_XIV	6
LG_XIV	C3_16	Alt_19	1	54.6	23	2.5	LG_XIII	2
LG_XV	C3_17	Alt_9	3	92.4	36	2.6	LG_XVI	9
LG_XVI	C3_19	Alt_21	1	66.7	12	6.1	LG_XIV	2
LG_XVII	C3_20	Alt_15	3	44.5	11	4.5	LG_XI	3
**Total**			**76**	**1687.6**	**694**			**125**
Average			4.05	99.3	40.8	2.5		7.4

^1^ Alignment with the map published by Sonnante et al. [21] and based on a cross between a globe artichoke (“Mola”) with a wild cardoon (“Tolfa”) genotypes.

The consensus map, obtained from the domesticated *C. cardunculus* forms, was compared with the Sonnante et al. [21] map constructed from a cross between the var. *scolymus* cultivar ‘Mola’ and the var. *sylvestris* (wild cardoon) accession ‘Tolfa’, by considering 125 (117 SSRs, eight SNPs) common markers. The common markers identified each of the 17 LGs on the consensus map, with between two and 21 present on each LG (Table 2). Ten of the LGs aligned readily; LGs V and VII aligned with two ‘Mola’/‘Tolfa’ LGs, and LG VIII with two major groups and a triplet of markers. LGs X/XVII, and XIII/XVI each aligned with only a single ‘Mola’/‘Tolfa’ LG. In general, marker order and genetic separation were comparable, with some exceptions. It has been established that wild cardoon is more divergent from the two cultivated forms (globe artichoke and cultivated cardoon) than are the two cultivated forms with respect to one another [44,45]. Somewhat surprisingly, therefore, over 100 SSR loci featured in the consensus map but apparently were either non-informative or remained as singlet loci in the ‘Mola’/‘Tolfa’ population.

### EST-SSRs as functional markers

Putative functions can be deduced for markers derived from ESTs using homology searches with public protein databases. Annotation of mapped loci was performed via BlastX search as well as InterPro scan and GO categorisation made it possible to tag some biological functions.

A set of 17 CyEM markers were annotated with GO terms involved in the ‘response to stimulus’ (Table 3), five of which were derived from transcripts related to ‘response to cold stress’ and eight to ‘response to salt stress’ terms. In particular, the marker CyEM-42, developed from the contig CL4773Contig1 (1281 bp, 267 aminoacids) [22] and mapped on LG_12 of “Romanesco C3” map, showed high amino acid similarity (81%) with the Arabidopsis protein kinase PBS1 (NP_196820.1, unigene At.23518). To consider reliable orthology, a reciprocal tblastx analysis against the whole EST collection, currently available for *C. cardunculus*, was performed and no better alignment than that of contig CL4773 was detected. PBS1 was found to work as R gene against the bacterial pathogen *Pseudomonas syringae*, where its cleavage, operated by the pathogens’ effector AvrPphB, triggers the signalling cascade, generating the host response (HR) [46]. *Pseudomonas* spp. together with other endophytic bacteria may affect globe artichoke plants both in field and during micropropagation [47] and the CyEM-42 may be likely considered a reliable marker for tagging a bacterial resistance trait in the species.

**Table 3:** CyEM markers with Gene Ontology annotation for stimuli response-related terms

**GO ID**	**Term**	**Level**	**N° of loci**	**Locus Name**
GO:0050896	response to stimulus	2	17	CyEM-008, CyEM-030, CyEM-42, CyEM-054, CyEM-057, CyEM-070, CyEM-072, CyEM-093, CyEM-120, CyEM-135, CyEM-145, CyEM-150, CyEM-152, CyEM-218, CyEM-229, CyEM-259, CyEM-266
GO:0009628	response to abiotic stimulus	3	13	CyEM-008, CyEM-030, CyEM-054, CyEM-070, CyEM-093, CyEM-120, CyEM-135, CyEM-145, CyEM-150, CyEM-152, CyEM-218, CyEM-229, CyEM-259
GO:0042221	response to chemical stimulus	3	4	CyEM-093, CyEM-218, CyEM-229, CyEM-266
GO:0006950	response to stress	3	15	CyEM-008, CyEM-030, CyEM-054, CyEM-057, CyEM-070, CyEM-072, CyEM-093, CyEM-120, CyEM-135, CyEM-145, CyEM-150, CyEM-152, CyEM-229, CyEM-259, CyEM-266
GO:0009266	response to temperature stimulus	4	5	CyEM-008, CyEM-054, CyEM-093, CyEM-145, CyEM-150
GO:0006970	response to osmotic stress	4	8	CyEM-030, CyEM-070, CyEM-093, CyEM-120, CyEM-135, CyEM-152, CyEM-229, CyEM-259
GO:0010033	response to organic substance	4	3	CyEM-093, CyEM-229, CyEM-266
GO:0009409	response to cold	4	5	CyEM-008, CyEM-054, CyEM-093, CyEM-145, CyEM-150
GO:0009651	response to salt stress	5	8	CyEM-030, CyEM-070, CyEM-093, CyEM-120, CyEM-135, CyEM-152, CyEM-229, CyEM-259

Redundancy occurs since each available GO-level of annotation was considered.

Our EST-SSR markers may be defined as functional markers with the potential to target polymorphisms in gene responsible for traits of interest and they can be also particularly useful for constructing comparative framework maps with other Asteraceae, giving the possibility to amplify ortholog genes and provide anchor loci.

### The genetic basis of earliness

**Figure 5:**
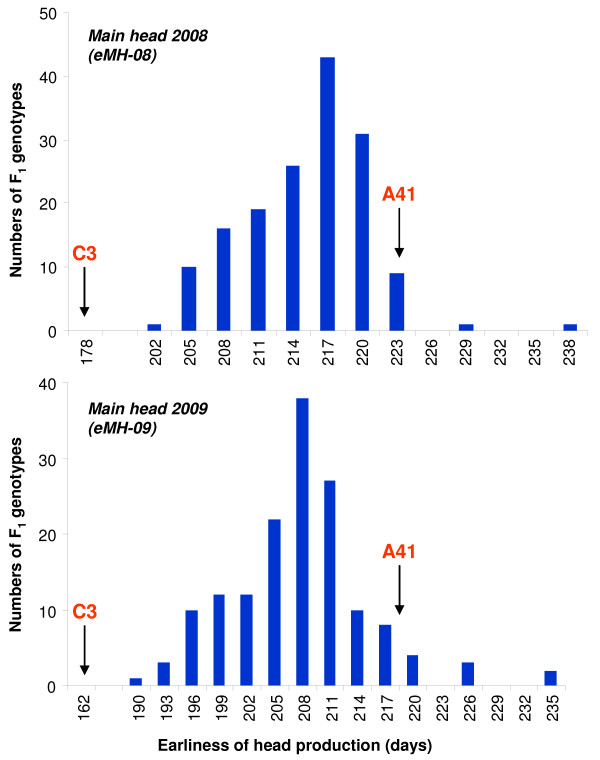
**Distribution of the harvesting time of the main head among 154 F**_**1**_**individuals in “2008” and “2009”.** Parental means (‘C3’: ‘Romanesco C3’, A41: ‘Altilis 41’) indicated by arrows.

An evaluation of the variance for the three earliness-related traits established significant genotypic differences (*P* < 0.05) between ‘Romanesco C3’ and ‘Altilis 41’ (Table 4). Thus, eMH in the former was 162 days in “2009” and 178 days in “2008”, while in the latter the respective times were 218 and 223 days. All three traits varied continuously among the F1 progeny (the distribution for eMH is shown in Figure 5); no progeny was as early flowering as ‘Romanesco C3’, but a few were later flowering than ‘Altilis 41’, due to transgressive segregation. The mean eMH, eFOH and eSOH lay substantially above the mid-parent value, suggesting semi-dominance for lateness. The low global level of heterozygosity characteristic of the cultivated cardoon makes it possible that one or more of the earliness QTL are in the homozygous state in ‘Altilis 41’, so that the presence of dominant alleles for lateness may contribute to later flowering across the whole mapping population. The inter-trait correlations were similar in both seasons, with the strongest correlation linking eMH and eFOH (r > 0.80, P < 0.0001). The correlations between the two seasons were also strong, ranging from 0.64 (P < 0.0001) for eMH to 0.49 (p < 0.001) for eSOH (Table 5). Flowering and head harvesting time was a little earlier in “2009” than in “2008” (7–8 days on average), while performance was somewhat more variable in “2009” (Table 4), probably reflecting the difference between re-awakened and newly sown material. The broad sense heritability for eMH of 0.76 (Table 4) indicated the trait to be predominantly under genetic control, but the rather lower heritabilities shown by the traits eFOH and eSOH suggested that the environment is quite influential in their determination.

**Table 4:** Earliness of the parental lines (‘C3’: ‘Romanesco C3’, ‘Alt 41’: ‘Altilis 41’) and their F1 progeny in “2008” and “2009”

**Precocity trait**	**Year**	**Parents**^**1**^		**F**_**1**_**population**^**2**^			
		**C3**	**A41**	**Mean**	**Range**	**s.e.**	**h**_**B**_^**2**^
Main head (eMH)	2008	178 a	223 b	212.9	202-238	0.518	0.76
	2009	162 a	218 b	206.1	190-235	0.694	
First order heads (eFOH)	2008	185 a	229 b	221.3	209-248	0.456	0.61
	2009	180 a	224 b	214.0	198-239	0.828	
Second order heads (eSOH)	2008	198 a	239 b	232.6	214-267	0.695	0.54
	2009	192 a	237 b	225.7	207-246	0.897	

^1^ Means followed by different letters within the same row are significantly different at *P* < 0.05.

^2^ s.e. : standard errors; h_B_^2^: broad sense heritability based on two years’ data.

**Table 5:** Correlations between the three earliness traits measured in the ‘Romanesco C3’ x ‘Altilis 41’ F1 population in “2008” and “2009”

**Precocity trait**	**Year**	**eMH**	**eFOH**	**eSOH**
**eMH**	2008	0.64***	0.84***	0.69***
	2009		0.86***	0.76***
**eFOH**	2008		0.54***	0.66***
	2009			0.72***
**eSOH**	2008			0.49**
	2009			

**P < 0.001; ***P < 0.0001.

**Figure 6:**
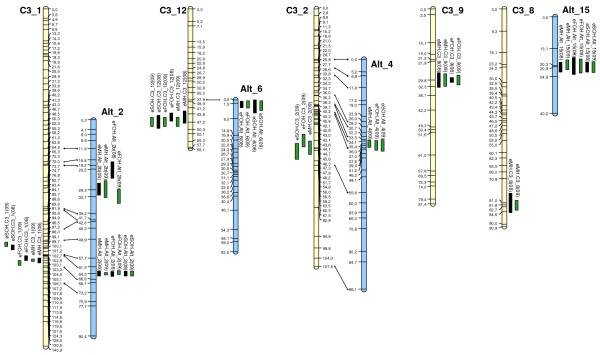
**Location of earliness QTL for the main head (MH), the first order head (FOH) and the second order head (SOH).** Only those LGs (‘Romanesco C3’ LGs shown in yellow, ‘Altilis 41’ ones in blue) harbouring QTL are shown. Black and green bars represent 1-LOD support intervals for each QTL detected in, respectively, “2008” and “2009”.

The KW test and SIM procedure identified, at first, six QTL regions stable across years in the developed consensus map (Figure 4). Those on LGs I, XI and XVII involved all three traits, those on LGs I and IX only eMH and eFOH, and the one on LG VII solely eMH. The seventh QTL cluster on LG II involved all three traits, but was only expressed in “2009” (Figure 4). On the whole, seven chromosomal regions scattered over six LGs of the consensus map were identified. When the ‘Romanesco C3’ and ‘Altilis 41’ maps were used separately for QTL validation, the percentage of phenotypic variance explained by some of the QTL differed from that predicted by the analysis based on the consensus map (data not shown), perhaps reflecting the structure and size of the segregating progeny and the existence of different allelic interactions [48]. However, all seven QTL regions were detectable by applying the SIM method to the parental maps, and further analysed with the MQM procedure. QTL identified in each map and season are shown in Table 6 and graphically reported in Figure 6. Only three of the seven QTL regions were detectable in both parental maps, presumably these regions were heterozygous in both parental lines. The other four were only detectable in one of the two maps, suggesting that one parent was homozygous in the critical region (Figure 6). Across all three traits, a total of 25 QTL was detected, of which 19 were stable across both growing seasons, with the other six expressed only in “2009”.

**Table 6:** Characteristics of the earliness QTL detected using the ‘Romanesco C3’ (C3) and the ‘Altilis 41’ (A41) maps

**Precocity trait**	**Map**	**LG**	**QTL**	**2008**						**2009**					
				**GW**	**Locus**	**cM**	**LOD**	**% var**	**Add**	**GW**	**Locus**	**cM**	**LOD**	**% var**	**Add**
Main head (eMH)	C3	C3_1	eMH.C3_1	3.6	rCELMS-40	105.1	14.7	47.9	−10.5	3.3	rCELMS-40	105.1	9.62	38.1	−11.6
		C3_9	eMH.C3_9		CyEM_10	29.8	4.8	10.5	−4.5		CyEM_10	29.8	3.4	7.6	−4.0
		C3_8	eMH.C3_8		CyEM_173	81.0	4.1	8.9	−4.3		CyEM_2	81.8	3.6	6.8	−3.5
		C3_12	eMH.C3_12		e39/m50-690	41.2	3.6	6.6	−3.6		e35/m50-302	47.2	3.3	6.1	−3.1
		C3_2	eMH.C3_2		-	-	-	-	-		e32/t81-380	58	3.9	13	6.4
	A41	Alt_2	eMH.Alt_2	3.3	e38/m47-158	64.3	6.2	41.4	−8.9	3.2	e38/m47-158	64.3	7.9	32.9	−10.9
		Alt_2	eMH.Alt_2b		p12/m62-150	29.3	3.6	10.7	−5.3		p12/m62-150	29.3	3.3	8.9	−5.7
		Alt_15	eMH.Alt_15		CyEM_248	20.9	3.3	8.9	4.2		CyEM_144	20.3	3.2	7.1	5.1
		Alt_4	eMH.Alt_4		-	-	-	-	-		e32/t82-90	35.4	3.4	9.8	5.2
First order head (eFOH)	C3	C3_1	eFOH.C3_1	3.4	e33/t89-430	104.0	8.9	31.0	−7.1	3.1	rCyEM_223	106.1	7.6	29.9	−10.5
		C3_9	eFOH.C3_9		CyEM_10	29.8	3.8	8.1	−4.1		CyEM_10	29.8	3.5	6.6	−5.1
		C3_12	eFOH.C3_12		e39/m50-690	41.2	3.4	5.6	−3.1		e35/m50-302	47.2	3.1	5.5	−4.8
		C3_2	eFOH.C3_2		-	-	-	-	-		e42/m50-176	55.0	3.2	5.1	−4.3
	A41	Alt_2	eFOH.Alt_2	3.0	e38/m47-158	64.3	7.2	25.6	−6.7	3.0	e38/m47-158	64.3	6.3	26.7	−10.2
		Alt_2	eFOH.Alt_2b		aCyEM_12	20.5	3.6	11.0	3.8		p12/m62-164	32.1	3.4	8.3	−5.4
		Alt_15	eFOH.Alt_15		CyEM_248	20.9	3.2	9.1	3.3		CyEM_144	20.3	3.6	7.8	5.3
		Alt_6	eFOH.Alt_6		cyre5/m47-160	1.5	3.0	8.3	3.1		cyre5/m47-160	1.5	3.0	5.7	3.4
		Alt_4	eFOH.Alt_4		-	-	-	-	-		e33/t89-490	36.0	3.0	6.3	4.2
Second order head (eSOH)	C3	C3_1	eSOH.C3_1	3.4	e38/t80-630	99.7	7.1	32.2	−9.7	3.2	e32/t81-98	98.0	5.6	22.4	−9.8
		C3_12	eSOH.C3_12		e35/m50-302	47.2	3.4	15.1	−7.1		e35/m50-302	47.2	3.4	18.0	−9.2
		C3_2	eSOH.C3_2		-	-	-	-	-		e38/m50-108	59.2	3.2	7.8	5.2
	A41	Alt_2	eSOH.Alt_2	3.1	e38/m47-158	64.3	4.9	19.0	−7.6	3.2	e38/m47-158	64.3	6.3	21.4	−8.2
		Alt_15	eSOH.Alt_15		CyEM_248	20.9	3.1	5.9	3.1		CyEM_144	20.3	3.5	9.1	6.4
		Alt_6	eSOH.Alt_6		cyre5/m47-160	1.5	3.1	7.4	3.9		cyre5/m47-160	1.5	3.2	6.1	4.1
		Alt_4	eSOH.Alt_4		-	-	-	-	-		e33/t89-490	36.0	3.2	5.9	4.1

Each QTL name is formed by the abbreviated form of the trait followed by the relevant LG. The table indicates genome-wide LOD thresholds (GW) as determined by a permutation test at *P* ≤ 0.05, the closest linked markers (Locus) and their map location (cM), the estimated LODs at the QTL peak (LOD), the proportions of the total variance explained (% var), and the additive effects (Add).

With respect to eMH, two of the QTL were heterozygous in both parents, three only in ‘Romanesco C3’ and two just in ‘Altilis 41’. The largest effect stable eMH QTL in ‘Romanesco C3’ mapped to LG C3_1 in the neighbourhood of the SSR locus CELMS_40, named eMH.C3_1. This QTL was responsible for 38-48% of the phenotypic variation and was associated with an additive effect of 10–12 days. The other four QTL in ‘Romanesco C3’ mapped to LGs C3_9, _8, _12 and _2, and accounted individually for between 6-10% of the phenotypic variance; eMH.C3_2 was only detected in “2009”. The largest stable QTL detected in ‘Altilis 41’ (eMH.Alt_2), homologous to the ones detected in the same region of the ‘C3’ map, explained from 33-41% of the phenotypic variance and was associated with an additive effect of 9–11 days. A second QTL, eMH.Alt_4, was detectable only in “2009”, but its location suggested it to be identical with eMH.C3_2 (Figure 6). Further two minor QTL present on LGs Alt_2 and _15 accounted for, respectively, 8% and-11% of the variance. Globally, the QTL identified in the ‘Romanesco C3’ and ‘Altilis 41’ maps accounted for, respectively, 74% and 62% of the phenotypic variance for main head harvesting time in “2008”.

Six eFOH QTL were detected, three of which were represented in both parental maps, one on just the ‘Romanesco C3’ map, and the other two on just the ‘Altilis 41’ map. Five of the six QTL mapped to a region where a eMH QTL was also located, with overlapping LOD confidence intervals but with an overall lower phenotypic effect. The exception was eFOH.Alt_6 (Table 6, Figure 6). As for eMH, the largest effect QTL mapped to LGs C3_1 and Alt_2 in the neighbourhood of CyEM_223. Based on the “2009” data, the set of eFOH QTL accounted for 47% (‘Romanesco C3’) and 54% (‘Altilis 41’) of the variation.

Only four eSOH QTL were uncovered, due to the reduced heritability of this trait (h_B_^2^ = 0.54, Table 4). All four co-localized with eFOH QTLs, with an overall lower phenotypic effect (Table 6, Figure 6), with the largest effect QTL mapping to the cluster on C3_1 and Alt_2. Based on the “2009” data, the set of eSOH QTL accounted for 48% (‘Romanesco C3’) and 43% (‘Altilis 41’) of the variation.

## Conclusions

We have reported here an extension of the *C. cardunculus* genetic map by introducing SSR loci sited within genic sequence. The integration of 139 of these loci has significantly improved the resolution and accuracy of the maps. Given that the genome size of the species is ~1.08Gbp [49], the mean equivalence between the physical and genetic length in this species is of the order of 1 cM to 670 kbp. Thus the mean physical separation of the mapped markers is around 2.2Mbp. On this basis, most gene sequences should lie within about 1Mbp of the nearest marker, although this value makes the non-valid assumption that recombination sites are randomly distributed along the length of the chromosomes.

Shortening the life cycle is seen as an important breeding goal in terms of both globe artichoke’s economic value, and the ease of exploiting cultivated cardoon as an energy crop [13,14]. The newly developed consensus as well as the parental genetic maps can accelerate the process of tagging and eventually isolating the genes underlying earliness in both the domesticated *C. cardunculus* forms. We have shown that a cluster of large effect QTL resides on the homologous LGs C3_1 and Alt_2, and this clearly represents a reasonable candidate for marker assisted breeding. The critical genetic interval contains two SSR loci (CELMS_40 and CyEM_223), either of which is well-placed to serve as an indirect selection criterion for earliness. Before such a genotypic selection programme can be implemented, however, a validation of the presence and importance of the QTL needs to be conducted using different genetic backgrounds and in other relevant environments [50,51]. To date, this study represents the first attempt to identify QTL in *C. cardunculus*, which is the necessary preliminary step for implementing marker-assisted selection for quantitative traits. Beyond tagging, mapping also prepares the ground for positional cloning, which will enable the molecular basis of trait variation to be identified.

## Methods

### Plant material and SSR analysis

The 178 informative CyEM markers identified by Scaglione et al. [22] were used to genotype a set of 94 F_1_ hybrid (randomly selected from 154 true hybrids as described by Portis et al. [17] from the cross’Romanesco C3’(globe artichoke; female parent) x ‘Altilis 41’ (cultivated cardoon; male parent). A 7 ng aliquot of genomic DNA from each mapping population individual was amplified in a 10 μl reaction containing 1x PCR buffer, 1 mM MgCl_2_, 0.5U Taq DNA polymerase (Qiagen Inc., Venlo, Netherlands), 40nM 5’-labelled (FAM, HEX or TAMRA) forward primer, 40 nM unlabelled reverse primer and 0.2 mM dNTP. A touchdown cycling regime was applied, consisting of an initial denaturation of 94°C/2.5 min, followed by nine cycles of 94°C/30s, 63°C/30s (decreasing by 0.7°C per cycle), 72°C/60s, and 30 further cycles of 94°C/30s, 57°C/30s, 72°C/60s. Where only weak amplification was achieved, the MgCl_2_ concentration was raised to 1.5 mM and the final annealing temperature was lowered to 55°C. The amplicons were separated on an ABI3730 capillary DNA sequencer (Applied Biosystem Inc., Foster City, CA, USA). Internal ROX-labelled GS500 size standards were included in each capillary. The output was analysed by GeneMapper v3.5 software (Applied Biosystems).

### Linkage analysis and parental maps construction

The CyEM genotypes of the 94 mapping population individuals were combined with previous genotypic data based on 605 AFLP, 27 S-SAP and 56 other SSRs [17], along with ten SNP from genes underlying caffeoylquinic acid synthesis (reported by Comino et al. [18] and Menin et al. [19]). JoinMap v4.0 [52] was used to generate two separate linkage maps (one for each parent) using the double pseudo-testcross mapping strategy [53]. The markers fell into three classes: maternal testcross markers segregating only in ‘Romanesco C3’ (expected segregation ratio 1:1); paternal testcross markers segregating only in ‘Altilis 41’ (1:1); and intercross markers segregating within both parents (either 1:2:1 or 1:1:1:1). Differences between observed and expected segregation ratios were tested by *χ*^2^, and only markers deviating if at all only slightly from expectation (χ_α=0.1_^2^ < *χ*^2^ ≤ χ_α=0.01_^2^) were used for map construction and the estimation of genetic distances, when their presence did not alter surrounding marker order in the LG. Heavily distorted loci (*χ*^2^ > χ_α=0.01_^2^), along with those associated with 30 or more missing values, were excluded. LGs were established on the basis of an initial LOD threshold of 6.0. Locus order and distances between loci were established using the following parameter set: Rec = 0.40, LOD = 1.0, Jump = 5. Map distances were converted to cM using the Kosambi mapping function [54]. Where a locus order discrepancy arose between a pair of parental LGs, the marker order of the ‘1:1:1:1’ segregating SSR and the marker order of SNP markers were taken as the ‘fixed order’. Once the framework maps had been established, additional loci were subsequently added and some LGs merged by lowering the LOD threshold to 5.0. On the resulting maps, loci suffering from slight segregation distortion have been identified with either one (χ_α=0.1_^2^ < *χ*^2^ ≤ χ_α=0.05_^2^) or two (χ_α=0.05_^2^ < *χ*^2^ ≤ χ_α=0.01_^2^) asterisks. The ‘Romanesco C3’ LGs are labelled LG_C3, and the ‘Altilis 41’ ones LG_Alt, using the numbering system suggested by Portis et al. [17].

### Consensus map construction

A genotypic data set based on all the available markers was then used to construct a consensus map. Here, the loci belonging to two segregation classes ‘1:2:1’ (the same pair of alleles segregating in each parent), and ‘1:1:1:1’ (different alleles segregating in each parent) were used as ‘bridge markers’. The most likely locus order was established from a comparison of the ‘C3’, ‘Alt’ and consensus LGs, and where these differed substantially from one another, the most likely order was assumed to be one associated with the lowest *χ*^2^ value (estimating goodness-of-fit) and the lowest mean *χ*^2^ contribution for all loci. LGs were established on the basis of an initial LOD threshold of 5.0 and numbered according to C3 maps LGs order.

### Sequence annotation

Annotation of mapped sequences was carried out using the Blast2Go software [55]. The best twenty BlastX results were retrieved by querying the nr protein database at NCBI. Gene Ontology terms were retrieved accordingly to software capabilities and transferred to our sequences, adopting an annotation threshold of 55. Additional GO terms were obtained performing InterPro scan for conserved motif, using all available databases. ANNEX function was used to obtain further GO terms which are implicit on the base of electronically annotated ones.

### Earliness evaluation and QTL analysis

The mapping population (154 F_1_ progeny of the cross ‘Romanesco C3’ (var. *scolymus*) x ‘Altilis 41’ (var. *altilis*)), along with six clones of each parental line, was cultivated at the University of Catania's experimental station (37°25’N; 15°30’E; 10 m a.s.l) and evaluated over the two growing seasons 2007–2008 (hereafter referred to as “2008”) and 2008–2009 (“2009”). In “2008”, seedlings at the three true leaf stages (about 40 days after germination) were transferred to the field in mid September, while in “2009”, the growing season was initiated by applying drip irrigation to field capacity in mid August. Earliness was scored either as the number of days between transplanting (“2008”) or awakening (“2009”) and harvesting of both the main (eMH trait) and first and second order heads (obtained from the ramification of the main stem: eFOH and eSOH traits). Population means, standard deviations, distribution histograms and trait correlations were calculated using R software [56]. Analyses of variance were based on treating each growing season as an independent replicate. The broad sense heritability was given by the expression h_B_^2^ = σ_g_^2^/(σ_g_^2^ + σ_e_^2^/y), where σ_g_^2^ represented the genetic variance and σ_e_^2^ the error variance. Correlations between traits were estimated using Pearson’s coefficient.

In the initial step of the QTL analysis, the consensus map was used to assign putative locations by performing a Kruskal-Wallis (KW) non-parametric test in conjunction with a simple interval mapping procedure (SIM) [57], applying the cross-pollination algorithm implemented within MapQTL v4.0 software [58]. Next, the two separate parental maps were employed for a re-analysis based on the BC1 algorithm, using both SIM and multiple QTL mapping (MQM) [59]. Markers lying within a putative QTL region and associated with the highest LOD score were used as co-factors. For the MQM, a backward elimination procedure was applied to select the appropriate co-factors (significantly associated with each trait at P < 0.02). The LOD thresholds for QTL significance were confirmed by a permutation test consisting of 1,000 replications, which implies a genome-wide significance level of 0.05 [60]. Only those QTL associated with a LOD greater than either the genome-wide threshold or the LG threshold were considered. 1-LOD support intervals were determined for each LOD peak [61]. The additive effect and the proportion of the overall phenotypic variance associated with each QTL and all QTL together were estimated from the MQM model. Linkage maps and QTL position were drawn using MapChart [62].

## Competing interests

The authors declare that they have no competing interests.

## Authors' contributions

SL and SK planned and supervised the experimental work; GM and RM cultivated the *C. cardunculus* parental accessions and the F_1_ individuals and performed earliness trait evaluation over the two growing seasons; DS and AA performed the genotyping of the progenies; EP and DS carried out linkage analyses and map construction; EP performed QTL analysis; SL and EP supervised the drafting of the manuscript. All authors read and approved the final manuscript.
